# 氟苯达唑抑制A549、H460细胞增殖并促进自噬

**DOI:** 10.3779/j.issn.1009-3419.2020.104.17

**Published:** 2020-05-20

**Authors:** 庭君 董, 泽军 路, 婧姣 李, 咏真 刘, 居一 温

**Affiliations:** 1 510280 广州, 南方医科大学第二临床医学院 The Second School of Clinical Medicine, Southern Medical University, Guangzhou 510280, China; 2 100048 北京, 解放军总医院第六医学中心肿瘤诊疗中心 Tumor Diagnosis and Treatment Center, The Sixth Medical Center of PLA General Hospital, Beijing 100048, China

**Keywords:** 氟苯达唑, 肺肿瘤, 自噬, Flubendazole, Lung neoplasms, Autophagy

## Abstract

**背景与目的:**

氟苯达唑是一种苯并咪唑类驱虫药, 既往研究发现其对结肠癌、乳腺癌细胞增殖具有抑制作用。本研究旨在探讨氟苯达唑对非小细胞肺癌A549、H460细胞增殖的影响及机制。

**方法:**

通过CCK-8(Cell Counting Kit-8)法检测不同浓度的氟苯达唑对A549、H460细胞活力的影响; Western blot法检测氟苯达唑处理后细胞自噬相关蛋白p62、LC3的表达水平; 自噬双标腺病毒(mRFP-GFP-LC3)转染细胞, 分析细胞内自噬流变化。

**结果:**

氟苯达唑抑制A549、H460细胞增殖, 并呈剂量依赖关系(*P* < 0.001)。2 μmol/L氟苯达唑处理A549、H460细胞24 h、48 h后p62减少, LC3 II/I比值升高(*P* < 0.005)。mRFP-GFP-LC3转染细胞显示氟苯达唑处理组红色荧光增加, 提示自噬流增强。

**结论:**

氟苯达唑可以抑制A549、H460细胞增殖并促进自噬。

## Abstract (en)

**Background and objective:**

Flubendazole is an anthelmintic and categorized in benzimidazole. Previous evidence indicates its suppression on proliferation of colon cancer and breast cancer cells. Our study aims to explore the effects of flubendazole on non-small cell lung cancer A549 and H460 cell lines and the underlying mechanism.

**Methods:**

CCK-8 assay was used to detect the effect of flubendazole at different concentrations on viability of both cell lines A549 and H460. We used western blot to detect the expression levels of autophagy-related proteins p62 and LC3 after flubendazole treatment. Cells were transfected with tandem fluorescent adenovirus (mRFP-GFP-LC3), and the impact of flubendazole treatment on autophagic flux were analyzed.

**Results:**

Cell viability analysis showed a dose-dependent inhibitory effect on proliferation of both A549 and H460, comparing to cells without flubendazole treating (*P* < 0.001). Level of p62 decreased and LC3 II/I ratio increased in cells treated with 2 μmol/L flubendazole for 24 h and 48 h, compared to control groups (*P* < 0.005). Red fluorescence signals increased in mRFP-GFP-LC3 transfected cells after flubendazole treating, suggesting an elevation in autophagic flux.

**Conclusion:**

Flubendazole may inhibit the proliferation of A549 and H460 cells and promote autophagy.

肺癌是我国乃至全世界最常见的恶性肿瘤之一, 其中非小细胞肺癌(non-small cell lung cancer, NSCLC)占所有肺癌的85%, 是全球癌症相关死亡的主要原因^[1]^。在过去的20年中, 含铂的双药方案化疗是晚期NSCLC的主要治疗手段, 然而, 化疗对晚期NSCLC患者的缓解率也只有40%左右, 且伴有不同程度的毒副反应。近年来随着分子生物学和肿瘤免疫学研究的持续深入, NSCLC的治疗方式发生了很大的改变, 不再是单纯的细胞毒疗法, 而是针对患者的相关驱动基因突变, 选择个体化靶向疗法, 或者以程序性死亡受体1(programmed death 1, PD-1)/进展期程序性死亡受体-配体1(programmed cell death-ligand 1, PD-L1)抗体为主的免疫检查点抑制剂治疗^[2]^, 但获得性耐药及药物的治疗有效率低仍是困扰医患的重要问题。因此, 通过研究肿瘤的发生发展途径寻找新的治疗药物尤为重要。

氟苯达唑(flubendazole)属于苯并咪唑家族, 主要用于人和动物的肠道寄生虫及全身性蠕虫感染, 具有良好的驱虫效果^[3]^。氟苯达唑能通过与微管蛋白相结合, 抑制微管蛋白的聚合作用, 阻滞细胞周期, 从而影响细胞增殖、有丝分裂等, 最后导致寄生虫的破坏^[4]^。近年来研究报导发现, 氟苯达唑还在抗癌方面发挥作用, 包括阻滞肿瘤细胞周期, 抑制肿瘤干细胞、抑制肿瘤细胞增殖与生长等^[5-7]^。此外, 氟苯达唑还被发现能激活自噬。氟苯达唑被认为是活性氧(reactive oxygen species, ROS)的有效诱导剂, 并且可以激活自噬。Zhang等^[8]^利用分子对接模拟技术, 对美国食品药品监督管理局(Food and Drug Administration, FDA)批准的多种小分子药物进行虚拟筛选, 发现氟苯达唑具有最佳的抗肿瘤活性和自噬相关基因(autophagy-related gene, ATG)4B酶活性, 进一步的分子动力学模拟结果显示, 氟苯达唑与ATG4B蛋白结合良好, 可以诱导自噬发生, 对乳腺癌MDA-MB-231细胞具有抗增殖作用。此外, 还有研究者^[9]^发现, 氟苯达唑在结肠癌细胞中通过阻断信号传导和转录激活因子3(signal transducer and activator of transcription 3, STAT3)信号传导发挥抗肿瘤活性, 并且激活自噬及诱导凋亡。氟苯达唑与化疗药物5-氟尿嘧啶在结肠癌治疗中显示出协同作用。

本研究拟采用NSCLC A549、H460细胞为研究对象, 探讨氟苯达唑对NSCLC细胞增殖的影响及作用机制, 为临床上困扰医患的NSCLC治疗提供了一条新思路。

## 1 材料与方法

### 1.1 材料

人NSCLC A549细胞由实验室保存传代, H460细胞购于上海中乔新舟生物科技有限公司, 氟苯达唑、LC3抗体购于Sigma-Aldrich公司, 羟氯喹(hydroxychloroquine, HCQ)、雷帕霉素(rapamycin)购于TargetMol公司, 自噬双标腺病毒(HBAD-mRFP-GFP-LC3)购于上海汉恒生物科技公司, RPMI-1640、FBS购于Gibco公司, PBS、Cell Counting Kit-8、4%组织细胞固定液购于北京索莱宝科技有限公司, p62抗体购于Abcam公司, GAPDH抗体购于Abbkine公司, 山羊抗兔-HRP、山羊抗鼠-HRP购于北京中杉金桥生物有限公司, PVDF膜(0.2 μm)购于Millipore公司, ECL发光液购于Beyotime公司, 二氧化碳培养箱购于上海Heal Force公司, MuliSKAN MK3酶标仪购于Thermo公司, 化学发光成像仪、电泳仪购于Tanon公司, SDPTOP CX40荧光生物显微镜购于宁波舜宇仪器有限公司。

### 1.2 方法

#### 1.2.1 细胞培养

A549、H460用含10% FBS、1%青链霉素混合液的RPMI-1640完全培养基, 在37 ℃、5% CO_2_培养箱中静置培养。细胞贴壁生长良好, 每2天传代一次。当细胞生长到对数期时, 用胰蛋白酶-EDTA消化液处理细胞, 待胞质回缩、细胞变圆、细胞之间不再连接成片后用2倍胰酶用量的RPMI-1640完全培养基终止消化。1, 000 rpm离心3 min, 弃上清, 制备成细胞悬液进行下一步操作。

#### 1.2.2 CCK-8(Cell Counting Kit-8)法检测细胞活力

将密度5×10^3^/孔的A549、H460细胞接种到96孔板中, 每孔100 μL, 置于37 ℃、5% CO_2_培养箱中培养, 24 h细胞贴壁后, 加入不同浓度的氟苯达唑(0.062, 5 μmol/L、0.125 μmol/L、0.25 μmol/L、0.5 μmol/L、1 μmol/L、2 μmol/L、4 μmol/L)为实验组, 以不加氟苯达唑为对照组, 空白组只有培养基和CCK-8溶液, 不含细胞。在培养箱中继续培养48 h后, 每孔加入10 μL CCK-8溶液, 培养箱中避光孵育2 h, 用酶标仪测定450 nm处的吸光度值。每个浓度设6个复孔。计算：细胞活力(%)=[A(实验组)-A(空白组)] / [A(对照组)-A(空白组)] ×100%。

#### 1.2.3 Western blot检测相关蛋白表达

将A549、H460细胞按2×10^5^/mL接种到六孔板中, 每孔2 mL。根据CCK-8细胞毒性检测实验的结果, 选择浓度为2 μmol/L的氟苯达唑为实验组, 不加氟苯达唑为对照组, 分别处理细胞12 h、24 h、48 h后, 加入100 μL RIPA裂解液(含100×蛋白磷酸酶抑制剂混合物), 用刮刀收集细胞, 冰上裂解30 min后, 4 ℃、12, 000 rpm离心15 min, 取上清于EP管中, 用BCA蛋白定量试剂盒测定蛋白浓度。制胶, 分别取30 μg样品蛋白/孔上样, 电泳, 湿转60 min(4 ℃, 100 v), 含5%脱脂奶粉的TBST封闭2 h, 分别用LC3、p62、GAPDH一抗稀释后4 ℃孵育过夜, TBST洗膜3次后, 用辣根过氧化物酶标记的二抗室温摇床孵育1 h, TBST洗膜3次, ECL发光液显影, 化学发光成像仪曝光, Image J软件分析条带灰度值。

#### 1.2.4 腺病毒转染和活细胞荧光成像

为进一步分析细胞自噬流, 利用含mRFP-GFP-LC3串联荧光蛋白的腺病毒(HBAD-mRFP-GFP-LC3)瞬时转染A549、H460细胞。将细胞按1.5×10^4^/孔的数量接种到细胞爬片上, 培养24 h后细胞密度为50%-70%, 按MOI=20加入含串联荧光蛋白的腺病毒, 8 h后更换为RPMI-1640完全培养基。36 h后按实验分组分别更换培养基、加入药物, 实验分为5组, 分别是空白对照组、氟苯达唑组(浓度为2 μmol/L)、氟苯达唑+羟氯喹组(浓度分别为2 μmol/L和40 μmol/L)、羟氯喹组(浓度为40 μmol/L)和雷帕霉素组(浓度为100 nmol/L)。培养24 h后, 吸去旧培养基, PBS清洗3次, 每次5 min, 加入4%组织细胞固定液固定30 min, PBS清洗, 避光封片, 利用倒置荧光显微镜及Toup View 3.7软件拍摄照片。

### 1.3 统计分析

采用SPSS 22、GraphPad Prism 7软件分析数据并作图, 数据以均数±标准差表示, 所有实验均重复3次。两组间比较采用独立样本*t*检验, 多组间比较采用单因素方差分析(*one-way ANOVA*)评价实验组间的差异, 多个样本均数间的多重比较采用*Dunnett-t*检验, *P* < 0.05为差异有统计学意义。

## 2 结果

### 2.1 氟苯达唑抑制A549、H460细胞增殖

分别用0 μmol/L、0.062, 5 μmol/L、0.125 μmol/L、0.25 μmol/L、0.5 μmol/L、1 μmol/L、2 μmol/L、4 μmol/L氟苯达唑处理细胞48 h, 采用CCK-8法测定A549、H460细胞活力。氟苯达唑对A549细胞的半数抑制浓度为1.231 μmol/L, 95%CI为1.007 μmol/L-1.506 μmol/L, 对H460细胞的半数抑制浓度为0.647 μmol/L, 95%CI为0.568 μmol/L-0.737 μmol/L。结果显示, 不同浓度的氟苯达唑处理A549、H460细胞后细胞活力下降(图 1), 具统计学差异(A549细胞组*F*=95.067, *P* < 0.001, H460细胞组*F*=365.617, *P* < 0.001)。进一步采用*Dunnett-t*检验进行组内多重比较, 与对照组(0 μmol/L氟苯达唑)相比, 随着氟苯达唑浓度的增加, A549及H460细胞的抑制越明显, 具剂量依赖性。

**1:**
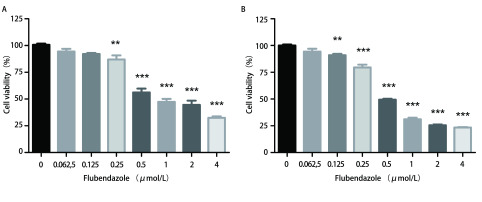
不同浓度氟苯达唑对A549(A)、H460(B)细胞增殖的影响。随着氟苯达唑浓度的增加, 细胞抑制越明显(**P < 0.01, ***P < 0.001)。 The effect of flubendazole at different concentrations on the proliferation of A549 (A) and H460 (B) cells. The inhibitory effect of flubendazole on proliferation elevated as the concentration increased (**P < 0.01, ***P < 0.001).

### 2.2 氟苯达唑促进A549、H460细胞自噬

选择浓度为2 μmol/L的氟苯达唑为实验组, 不加氟苯达唑为对照组, 分别处理细胞12 h、24 h、48 h, 并进行Western blot实验。在A549细胞中(图 2A-C), 和对照组相比, 24 h和48 h时实验组p62蛋白表达下降(*t*=17.82, *P* < 0.001; *t*=5.895, *P*=0.004), 12 h、24 h、48 h时LC3 II/I均升高(*t*=7.798, *P*=0.001; *t*=37.856, *P* < 0.001; *t*=17.725, *P* < 0.001)。在H460细胞中(图 2D-F), 和对照组相比, 12 h、24 h、48 h时实验组p62蛋白表达下降(*t*=14.481, *P* < 0.001; *t*=12.827, *P* < 0.001; *t*=21.825, *P* < 0.001), LC3 II/I比值升高(*t*=25.276, *P* < 0.001; *t*=17.787, *P* < 0.001; *t*=29.276, *P* < 0.001)。上述实验结果提示氟苯达唑促进A549、H460细胞自噬。

**2:**
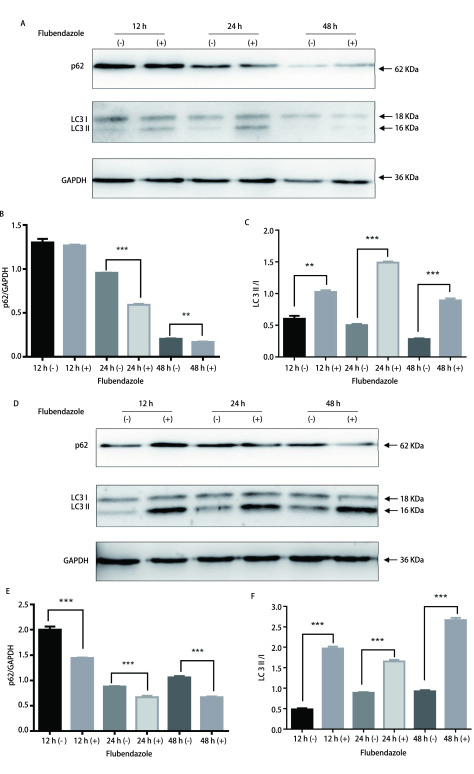
氟苯达唑在A549细胞(A-C)中对自噬相关蛋白的影响, B、C分别代表p62/GAPDH、LC3 II/I的灰度值分析。氟苯达唑在H460细胞(D-F)中对自噬相关蛋白的影响, E、F分别代表p62/GAPDH、LC3 II/I的灰度值分析(**P < 0.01, ***P < 0.001)。 The effect of flubendazole on autophagy-related proteins in A549 cells (A-C), B, C represents expression level of p62/GAPDH and LC3 II/I. Expression of autophagy-related proteins in H460 cells after flubendazole treatment (D-F), E, F represents expression of p62/GAPDH and LC3 II/I. Quantification were conducted through gray value analysis (**P < 0.01, ***P < 0.001).

### 2.3 氟苯达唑促进A549、H460细胞自噬流

我们使用含mRFP-GFP-LC3串联荧光蛋白的腺病毒对A549、H460细胞进行转染。实验分为5组, 分别是空白对照组、氟苯达唑组(浓度为2 μmol/L)、氟苯达唑+羟氯喹组(浓度分别为2 μmol/L和40 μmol/L)、羟氯喹组(浓度为40 μmol/L)和雷帕霉素组(浓度为100 nmol/L)。实验结果如图 3和图 4所示, 空白对照组中未见自噬体或较少, 且红色荧光与绿色荧光表达一致, 此时细胞自噬水平较低。加入氟苯达唑诱导后, 红色荧光增加, 绿色荧光较少, 融合(merge)后为红色、黄色点状荧光, 并且以红色荧光为主。氟苯达唑与羟氯喹共同作用时, 绿色荧光表达量较氟苯达唑组升高, 融合后可看见红色、黄色荧光相间, 并且黄色荧光较多。羟氯喹组红色荧光与绿色荧光表达一致, 融合后呈黄色荧光为主。雷帕霉素处理组可见细胞内有大量红色荧光斑点, 绿色斑点较少, 融合后以红色荧光为主。

**3:**
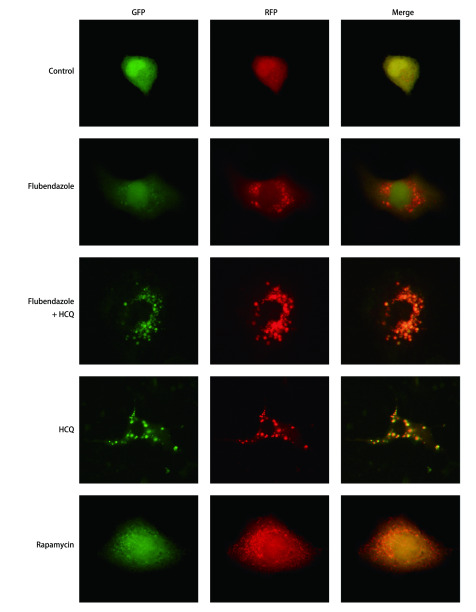
RFP-GFP-LC3转染的A549细胞中对照组、氟苯达唑组、氟苯达唑+羟氯喹组、羟氯喹组和雷帕霉素组自噬流变化(×400)。 Variations of autophagic flux in control group, flubendazole group, flubendazole+hydroxychloroquine group, hydroxychloroquine group and rapamycin group of RFP-GFP-LC3 transfected A549 cells (×400).

**4:**
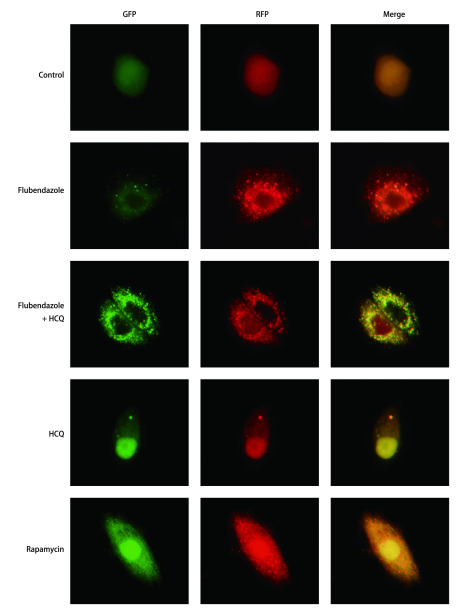
RFP-GFP-LC3转染的H460细胞中对照组、氟苯达唑组、氟苯达唑+羟氯喹组、羟氯喹组和雷帕霉素组自噬流变化(×400)。 Variations of autophagic flux in control group, flubendazole group, flubendazole+hydroxychloroquine group, hydroxychloroquine group and rapamycin group of RFP-GFP-LC3 transfected H460 cells (×400).

## 3 讨论

氟苯达唑是一种抗真菌药物, 近年来研究结果显示, 氟苯达唑是一种潜在的抗肿瘤药物, 可以抑制肿瘤细胞增殖, 包括骨髓瘤、白血病、神经胶质瘤、乳腺癌、结直肠癌、黑色素瘤、食管鳞癌等^[5, 9-13]^, 但目前并未对NSCLC进行研究。甲苯达唑也属于苯并咪唑家族, 两者在结构和功能上具有相似性, 并且对NSCLC具有抗肿瘤效应^[14]^。本研究通过CCK-8实验初步验证氟苯达唑对A549、H460细胞增殖的影响。结果表明, 不同浓度的氟苯达唑对A549、H460细胞增殖有抑制作用, 且随着浓度升高, 细胞活力逐渐下降。

自噬是将细胞溶质和/或其特定内容物递送至溶酶体以进行降解的细胞内分解代谢过程, 在肿瘤细胞中起着双刃剑的作用。一方面, 自噬使肿瘤细胞能够耐受包括缺氧微环境、饥饿和可能某些形式的治疗在内的压力。另一方面, 自噬在减少应激损伤、限制肿瘤基因发生中起重要作用, 通过清除受损的蛋白质和细胞器, 或者通过细胞内循环维持能量平衡, 自噬最终可以防止导致肿瘤发生的基因组损伤^[15]^。

微管相关蛋白轻链3(microtubule-associated protein 1 light chain 3, LC3)蛋白是衡量自噬水平的一个重要指标。自噬体形成过程中, LC3蛋白被ATG4蛋白切割成LC3 I, 在ATG3蛋白、ATG7蛋白的作用下与磷脂酰乙醇胺(PE)缀合形成LC3 II, 因此, 自噬活化时, 细胞内LC3 I向LC3 II转化, LC3 II/I比值升高。p62蛋白是选择性自噬受体, 在自噬过程中与泛素化蛋白结合, 最终被清除, 当自噬受到抑制时, 会导致p62堆积, 从而使p62蛋白表达升高。因此, 自噬相关蛋白表达水平能反映自噬水平的高低。本研究Western blot实验结果显示, 浓度为2 μmol/L的氟苯达唑处理A549、H460细胞后p62表达下降, LC3 II/I比值升高, 提示在这个过程中促进自噬的发生。

为进一步验证细胞内自噬流的变化, 研究人员用含自噬双标荧光蛋白的腺病毒转染细胞, 并在36 h后加入处理药物。其中, 羟氯喹是氯喹的类似物, 可以使pH升高, 阻断自噬体与溶酶体融合, 从而阻滞自噬的发生^[16]^, 为阴性对照。雷帕霉素是mTOR的抑制剂, 可以促进自噬的发生, 作阳性对照。在自噬早期, GFP发绿色荧光, RFP发红色荧光, 两者融合为黄色荧光。在自噬晚期, 自噬体与溶酶体融合后pH值降低, GFP淬灭, 融合后红色斑点增多, 提示自噬流水平增高。实验结果显示, 与对照组相比, 氟苯达唑组和雷帕霉素组中荧光融合后以红色荧光为主, 提示氟苯达唑与雷帕霉素作用相似, 促进自噬流活化。当氟苯达唑与羟氯喹共同作用时, 由于羟氯喹阻滞自噬体与溶酶体结合, 绿色荧光表达量较单用氟苯达唑组升高, 融合后可看见红色、黄色荧光相间, 并且黄色荧光较多。羟氯喹组红色荧光与绿色荧光表达一致, 融合后呈黄色荧光, 代表自噬流受到阻断。研究证明, 氟苯达唑可促进NSCLC细胞A549、H460自噬的发生。

综上所述, 我们发现氟苯达唑是一种潜在的肺癌化疗药, 能抑制NSCLC细胞的增殖, 降低A549、H460细胞活力。进一步研究发现氟苯达唑可上调LC3 II/I的比值, 减少p62蛋白, 促进A549、H460细胞自噬流活化, 诱导细胞自噬。氟苯达唑促进细胞自噬的具体信号通路还需进行进一步研究。通过此研究, 有望为寻找新型的NSCLC治疗药物提供了一定的基础研究证据支持。
